# Transactions of the Medical Society of the State of New York

**Published:** 1857-05

**Authors:** 


*Transactions of the Medical Society of the State of New York.* Published at Albany, Feb., 1857.

This is a volume of 292 pages, printed for the Society by the Legislature of the State of New York.

Its contents are of more than usual interest as they embrace the Proceedings of the Semi-Centennial Anniversary of the Society; fifty years having elapsed since its first organization. A large part of the volume is occupied by biographical sketches of physicians who lived and held prominent positions in the profession during the early part of the present century.

There is also a paper of some interest on Cholera Infantum and Cholera Morbus, by Dr. Ed. H. Parker, of New York city. But the paper which we read with most pleasure, was the Semi-Centennial Address by Dr. Alden March, the eminent professor of Surgery in the Albany Medical College. The whole address is appropriate in sentiment, and contains many passages of genuine eloquence and beauty. For the special benefit of such as are desponding, and disposed to esteem lightly the profession to which they belong, we quote a few of the concluding pages of the Address:—

" Formerly, towards the middle of the last century, fifty or sixty out of every hundred children born in London, died before they had reached their fifth year of age; but the mortality has gradually and steadily diminished, so that now not above thirty or thirty-five in every hundred die at that early period."

'At the present time there are more than 600,000 children born annually in Great Britain. According to the above scale of mortality, more than 300,000 of these would have perished formerly before they were five years old; now only about 200,000 die during the first five years of life: thus showing a saving of human life in this item alone, in the population of the British Isles, to the extent of at least 100,000 a year.

'By reference to the tables of "Vital Statistics," we farther learn, "that in the latter part of the sixteenth century one-half of all who were born died under five years of age; the average longevity of the whole population was but eighteen years. In the seventeenth century one-half of the population died under twelve years of age. But in the first sixty years of the eighteenth century one-half of the population lived over twenty-seven years. In the latter forty years one-half exceeded thirty-four years of age. At the beginning of the present century one-half exceeded forty years; and from 1838 to 1845 one-half exceeded forty-three years. The average longevity at these successive periods has been increased from 18 years in the sixteenth century, up to 43 7-10 by the last reports."

'There are certain classes of moral as well as physical defects and derangements that have of late years, and I am quite certain within the last half century, in this country at least, attracted the attention of the humane physician. And I think we may say with confidence and with pride, that no class of men, professional or otherwise, have done more to ameliorate the moral and physical condition of the unfortunate, the poor and degraded, and to aid the cause of religion, than our profession.

'Fifty years ago where were our medical and surgical hospitals, our houses of correction for juvenile delinquents, our insane hospitals, our asylums for the deaf and dumb, our retreat for the idiot, and, as is now contemplated, a refuge for the poor, besotted inebriate? My sensitive and sympathising audience hardly need to be excited with the recital of the dark picture of the condition of the unfortunate and unhappy maniac, previous to the commencement of the present century. Then his habitation was either a dark, narrow cell, or a cage; he was secured by a straight jacket, or manacled and chained with as much unfeeling severity as a galley slave; his treatment was starvation and filth, stripes and bruises, scorn and hatred. Now, the body and mind are cared for and scientifically treated. All these benevolent and praiseworthy institutions are under the superintendence of kind-hearted physicians.

'Let us next devote a few moments to the consideration of the agencies that have been employed during the last half century in enlarging the field of medical knowledge.

'At the present time, colleges and schools devoted to medical instruction, and hospitals managed by physicians and surgeons, afford the chief means for educating young men in the practice of physic and surgery.

'In 1807 there were not half-a-dozen medical schools in the United States; nor scarcely a greater number of hospitals. Now there are about forty medical colleges or associations where medicine and surgery are taught; and it is probable that there are twice that number of hospitals scattered through the cities and large towns of the United States.

'The names and dates of the organization of the five oldest medical institutions in this country are the following, viz: The Medical Department of the University of Pennsylvania was established in 1765. The College of Physicians and Surgeons of the city of New York was instituted 1768, in connection with Columbia College, and in 1807 obtained an independent charter. The Medical School of Boston, Mass., now the Medical Department of Harvard University, was organized in 1782. The Medical Department of Dartmouth College, in 1798; and the University of Maryland, Baltimore, in 1807.

'When our medical colleges or schools were limited to five, and the hospitals of this country but a little more numerous; when the whole apparatus, chemicals, and chemical tests of the chemist's laboratory could be almost packed in a bushel; when the anatomical museum of a college consisted of two or three smoky skeletons, a handful of disjointed bones, and a few coarsely injected preparations; and when a pathological cabinet was not known in our country: it requires no very great stretch of the imagination to draw the contrast between the advantage of 1807 and those of 1857, for obtaining a thorough practical medical education.

'We are now prepared to ask the question, what agency do medical societies exert in advancing and improving medical science? If the act to incorporate medical societies had been made to read, "for the purpose of extending and improving medical science," instead of simply, "for the purpose of regulating the practice of physic and surgery," it would have been more in conformity to the spirit and practical working of our society as it is now organized.

'In addressing the Medico-Chirurgical Society of Edinburgh, on the modern advancement of physic, Prof. Simpson says, "I believe that at the present time there exists but one opinion in relation to the fact, that the study of medicine, like the study of other departments of practical science and art, has been vastly promoted by associations like our own."

'Prof. Wood, late President of the American Medical Association, in his address before that body, in speaking of the tendency of the profession to become indolent, careless, and mercenary in protecting the best interests of the profession, says: "The medical mind, anterior to the birth of this association, was in a state of comparative inertia. In all the departments of the profession, the educational as well as the practical, material interests began to preponderate. The great struggle seemed to be in the teaching department to gather pupils; in the practical to gather patients: in both, to swell the pockets. No wonder that quackery loomed upwards, as regular medicine began to sink." He adds: "But the association arose, and a new spirit was awakened. Many had been watching this apparent abasement of the profession with sorrow; but they were powerless in their isolation. No sooner had the flag of the association been given to the breeze, than they hastened to join its standard. From all quarters and from the remotest bounds of the country, volunteers poured in to join this great crusade against the evils which had been usurping the sacred places of the profession. The mass of medical society was moved to its very depth. Hundreds upon hundreds came forth from their sheltering privacy, and threw their souls into the grand movement which was to conquer, to purify, and regenerate the prostrated glory of their calling. The feeble voice of opposition was heard for a moment; but it was soon drowned in the overwhelming shouts of the masses crying out, Onward! Onward!"

'Those who felt but little confidence in the anticipated good that was to arise from the organization of the American Medical Association, either to the public or to the profession, must acknowledge that a new impulse has been given to the science of medicine in this country since it was founded. And the glory of starting the ball is due to the State of New York—to this society; and almost entirely to the untired exertions of

Prof. N. S. Davis, now of Chicago, in the call of a convention, suggested by the writer, out of which the association grew. It appears on record that Dr. Hays, of Philadelphia, first suggested the idea of instituting a "National Medical Association;" and that the honor of submitting the plan for the permanent organization of the "American Medical Association," is due to John Watson, M.D., of New York, chairman of the committee of organization.

'Well may we be proud, not only of our own society and its achievements in literature and science, but also of the agency it has had in promoting the *national* prosperity of our noble profession. Let us then continue to act honestly and faithfully in the discharge of our professional duties to each other, and to the public, even if our motives should be impugned or fail to be properly appreciated. We may meet with discouragement, neglect and insult, but let us not weary in well doing.

'The storms and tempests of quackery may assault the citadel of the science of medicine and surgery. The good, old, and well-tried ship "Regular," commanded by officers and recruits of the orthodox profession of medicine, may be tossed and veered about by the popular "isms" of the day—nay, she may occasionally lose a *spar*, or receive a *breach* of continuity in some of her light cordage: yet her mainmast stands erect; without *fracture* of her beams, without *dislocation* of her helm, and with a hull as sound and as safe as on the day she was launched. She still floats on the sea of "Confidence," and even though she may now and then be threatened with a mutiny among some of her undisciplined recruits, yet we find the captain in command, the pilot at his post, the helmsman on duty, and the watchman at mast head, warning us of approaching danger.

'Rapid and imperfect as has been our sketch of the past, may we not see enough in it to fill us with high and encouraging hopes for the future? And, as we look forward through the vista of another half century, may we not confidently hope that our successors will be able, in their turn, to leave upon record an account of far greater and more numerous achievements in the arts and sciences and advancements in our profession, than has been our privilege to record.

'Our social relations, and the kindly feelings our annual re-unions are calculated to promote, are well fitted to exert a happy influence on our hearts, and to inspire confidence in and respect for each other.

'Let these sentiments be cherished, and whether it shall be our lot again to meet here, or hereafter, may it be one of serene and unalloyed enjoyment.'

